# Stress reduction interventions for patients with chronic diabetic foot ulcers: a qualitative study into patients and caregivers’ perceptions

**DOI:** 10.1186/s13047-022-00592-x

**Published:** 2023-02-06

**Authors:** M. Graça Pereira, Susana Pedras, André Louro, Alberto Lopes, Margarida Vilaça

**Affiliations:** 1 Psychology Research Centre (CIPsi), University of Minho, Braga, Portugal; 2 School of Psychology, Department of Applied Psychology, University of Minho, Campus de Gualtar, Braga, 4710-057 Portugal; 3 Angiology & Vascular Surgery Department, Centro Hospitalar Universitário Porto (CHUP), Porto, Portugal; 4 Health & Family Research Group, Psychology Research Centre (CIPsi), University of Minho, Braga, Portugal; 5 Portuguese Association of Clinical Hypnosis and Hypnoanalysis (APHCH), Porto, Portugal

**Keywords:** Diabetic foot ulcer, Hypnosis, Progressive muscle relaxation, Patients, Family caregivers

## Abstract

**Background:**

The present study aimed to assess the perceptions of patients with chronic diabetic foot ulcers (DFUs) and their family caregivers regarding the impact of two stress reduction interventions on DFU and psychological wellbeing. The intervention included progressive muscle relaxation and hypnosis sessions.

**Methods:**

This study used a qualitative exploratory design and included individual interviews with eight patients with chronic DFUs and six family caregivers, using a semi-structured interview guide. Transcript analysis employed thematic content analysis.

**Results:**

Four key themes common to patients and their caregivers were found: 1) perspectives regarding the intervention; 2) intervention effectiveness; 3) perceived importance of psychology in the DFU treatment; and 4) emotional consequences associated with DFUs. Although themes were common to both intervention groups, sub-themes from the last two themes differed for patients that received muscle relaxation versus those who received hypnosis.

**Conclusion:**

Patients and caregivers reported perceived benefits from both interventions, regarding DFU healing and emotional wellbeing. Patients who received hypnosis and their caregivers also reported lasting effects. Participants suggested that psychological interventions such as stress reduction interventions could be included in the DFU standard treatment as an adjuvant to the clinical protocol for DFU treatment, preferably offered early on, when patients begin treatment at the diabetic foot consultation.

## Background

Diabetes is a chronic systemic disease with a steadily increasing prevalence worldwide. There are currently 537 million people with diabetes mellitus (DM), of which around 61 million are living in Europe [1]. Portugal is one of the five European countries with high prevalence of people between the ages of 20 to 79 years with diabetes (9.1%) [1]. In Portugal, the annual health expenditures per patient with DM, including health services, family and nutrition activities, and emergency aid, is 2293.3 USD [1].

One of the most common, serious, and feared complications of people with DM is a diabetic foot ulcer (DFU), frequently resulting from poor glycemic control and repetitive trauma to a sensory or vascular compromised foot [2]. Over half of patients with DM, with foot injuries, will develop an infection, which may result in the amputation of the lower extremities, and ultimately, in death [3–5]. In fact, previous studies suggest a five-year survival rate for approximately half of patients with DFUs who undergo major or minor amputations [6]. Today, DFUs remain a public health problem, representing a considerable financial burden to health care systems and the society [7].

The high rates of disability and mortality in patients with DFUs cause a great burden to patients, their families, and society. Patients dealing with DFUs report a variety of physical and emotional difficulties. These include bodily pain, mobility limitations, dependence on others, increased health care needs, risk of amputation, decreased sociability, frustration, grief, anxiety, and depression, with an adverse impact on patients’ quality of life (QoL) [8, 9]. These difficulties further lead to significant changes in patients’ lifestyle, causing emotional distress. In fact, patients with DM are twice more likely to suffer from anxiety and depression than those without diabetes [10], and those with DFUs show a greater tendency to experience anxiety and depression compared to patients with DM but no wounds [11]. The prevalence of psychological morbidity is also larger in patients with DFUs that suffer from other diabetes complications [12].

At home, the treatment of chronic DFUs often requires the involvement of family members. Family or informal caregivers are often offspring or a spouse that provides unpaid support, with an important role in monitoring patients’ self-management, detecting improvements or deteriorations in the wound’s progression, as well as in providing daily care. Although family caregivers often feel unprepared to provide care, they accept their role mostly because of feelings of moral or social obligation [13], particularly in Portugal where traditionally the caregiver role is assigned to family members. Consequently, caregiver’s QoL declines, while the psychological burden increases over time [14–16]. Despite all challenges that family caregivers face, they undoubtedly play a crucial role in the recovery of patients with DFUs, having an important voice in patients’ recovery [17].

The prevention and management of DFUs is a major therapeutic challenge and concern for patients with diabetes, family caregivers, and health care professionals [17, 18]. Since DFUs tend to have a poor prognosis, and may become recurrent, taking weeks or months to heal [19], an integrated and multidisciplinary approach is crucial for a successful management of DFUs. Given the negative effect of psychological distress on wound healing, and overall health [20, 21], it is important to identify what support patients with DFUs may need to deal with the stress involved with this serious condition, and its impact on QoL. Psychological interventions, such as relaxation training techniques or hypnosis, have already shown positive results in the management of DM [22–24] and in patients with DFUs, [25], representing promising effective adjuvant interventions in the treatment of DFUs.

The aim of the present study was to capture the perspectives of clinically distressed patients and their family caregivers on the effectiveness of two stress reduction interventions towards DFU healing progression and psychological wellbeing. Specifically, this study addresses patients and informal caregivers’ perspectives on progressive muscle relaxation (with guided imagery) and hypnosis (with guided imagery).

## Methods

### Design and methods

This is a qualitative study nested in a larger longitudinal randomized controlled trial (RCT) study focused on the effectiveness of two stress reduction interventions in clinically distressed patients with chronic DFUs. The RCT is already registered in the ClinicalTrials.gov platform (Registration number: NCT04698720), and the study protocol is published elsewhere [26] and summarized below. The use of qualitative research methods is particularly valuable when studying complex health-related topics [27], such as the DFUs healing process, allowing a refinement of quantitative outcomes from previous findings of the RCT [26] throughout a more in-depth exploration. Furthermore, according to Kuhnke et al., [28] the qualitative approach allows a deeper understanding of the experience of living with a DFU, compared with other research methods.

Patients who finished  at least 75% of the stress reduction sessions, and completed the baseline (T0) and the post-intervention assessment approximately two months after (T1), were verbally informed by the researcher about the purpose of this study, and invited to participate. After obtaining written informed consent, individual face-to-face, semi-structured interviews were conducted approximately two weeks after completing the intervention. At the end of the interview, patients were asked if they agreed that the researcher contacted their family caregiver to inform, and invite them to participate in a similar interview. All study participants (patients and caregivers) signed written informed consent forms, and agreed to be audio-recorded during the interviews.

The interviews were conducted from December 2020 to November 2021, and ethical approval was obtained from the two hospitals where participants were recruited.

### Participants and recruitment

This exploratory qualitative study was conducted in two central hospitals with multidisciplinary diabetic foot clinics, in the North of Portugal. The two hospitals work independently, and both diabetic foot clinics are referral clinics in the DFU treatment. Participants were patients who participated in the larger RCT study [26]. Inclusion criteria for the RCT study were: i) adult patients with type 2 DM, ii) one or two chronic active DFUs (a non-healing ulcer for six or more weeks and less than 12 weeks) at the baseline evaluation, and iii) significant clinical stress, anxiety, or depression levels. Clinical distress was defined according to the Hospital Anxiety and Depression Scale (HADS) [29, 30] and the Perceived Stress Scale (PSS) [31, 32], with patients with scores higher than 11 on the HADS subscales, or higher than 13 (men) or 17 (women) on the PSS, evaluated as being clinically distressed. Exclusion criteria included patients i) with a recurrent DFU at the baseline e, ii) undergoing hemodialysis, iii) with a diagnosis of cancer, iv) with a history of mental illness, v) with dementia or unable to communicate, and vi) receiving psychological support during the study period. After the inclusion in the larger study, patients that i) completed at least three of the four intervention/control protocol sessions, and ii) were able to provide in-depth and rich-texture information regarding the intervention’s impact were selected to participate in the nested qualitative study. This sampling method was adopted since qualitative purposive sampling is considered to be more efficient than random sampling [33].

Initially, data collection was expected to include at least four single-per-participant interviews per group (progressive muscle relaxation versus hypnosis). However, given that two patients did not have a caregiver, the final sample included eight patients and six caregivers.

### Interventions

Four participants allocated in the treatment group 1 (TG1) completed progressive muscle relaxation with guided imagery intervention (PMR + GI), and three participants allocated in the treatment group 2 (TG2) completed hypnosis with guided imagery intervention (H + GI). One participant from the TG2 only received three sessions because the DFU healed before the fourth session.

PMR + GI sessions began with diaphragmatic breathing, followed by Jacobson’s progressive muscle relaxation, a technique that consists of consequently tensing and relaxing individual muscle groups of the body. After completing the relaxation exercises, guided imagery focused on the DFU healing was introduced. H + GI sessions followed a hypnotic protocol that included the following steps: presentation of the session’s goal and unconsciously call for change (pre-talk); capture of patients’ attention to absorb the sensations from the surrounding environment and/or the body (absorption); use of direct and indirect hypnotic suggestions (ratification); use of simple language patterns and hypnotic techniques to provide patients evidence that the hypnotic trance is happening (aliciation); activation of patients’ unconscious responses through hypnotic language and guide imagery (dissociation); and awakening of the patients’ hypnotic mindset (awakening).

Both interventions followed a script that included four sessions of 45 minutes duration each, to be implemented every two weeks, in approximately a 2-month treatment course. Sessions were conducted in a private room, with a specialist treatment couch, provided by the two hospitals were data collection took place.

### Data collection

#### Instruments

Patient’s sociodemographic information (e.g., gender, age, education, residence, marital and professional status) was collected at the baseline assessment (T0), using a sociodemographic questionnaire developed by the research team for this study. Clinical information was collected through a clinical questionnaire that assessed the detailed clinical history of patients. Clinical data was provided by the patients’ physician or nurse at T0, and included the type and duration of diabetes, glycemic control, other pre-existing complications of diabetes, diabetic foot type, DFU location and duration, previous DFUs, and concomitant treatment.

Caregiver’s sociodemographic information was gathered through a brief questionnaire that included questions regarding the caregivers’ age, residence, education, marital status, professional status, and years of caregiving. Caregivers answered this questionnaire before initiating the interview.

#### Interview

The interview guide was prepared by the research team based on: i) the literature review on the assessment of the effectiveness of  the two interventions, ii) the researchers’ experience with both interventions, and iii) the study goal. Questions explored patients and caregivers’ perceptions regarding the effectiveness of stress reduction sessions on patients’ wellbeing and DFU healing progression, whether directly or indirectly. Specifically, the interview addressed the perspectives of patients and caregivers on:

I) Expectations and thoughts regarding the stress reduction interventions;
II) The contribution of the stress reduction interventions towards patient’s wellbeing, and the DFU healing in particular.
III) The way the stress reduction intervention sessions were delivered;
IV) The importance for the multidisciplinary diabetic foot clinic to offer this type of intervention on a regular basis.

Two trained researchers with a PhD in Health Psychology conducted the interviews in a private room reserved by the hospitals to this study. The interview guide was used flexibly in order to follow the natural course of the participants’ discourse. Each interview was approximately 30 minutes in length. Interviews were audio-recorded and transcribed verbatim by the two researchers that conducted the interviews.

### Data analysis

Two authors trained in qualitative research methods used deductive thematic analysis to generate predominant themes and sub-themes [34]. To ensure reliability, the two researchers coded the transcripts of eight participants independently, and then met throughout the coding process to solve coding issues through consensus, thus ensuring agreement on themes derived from the data and interview guide. According to Miles and Huberman’s formula [35], inter-rater reliability was 0.84%, at this stage. Two more meetings were held during the coding process to discuss themes found in the remaining transcripts. Finally, the first author reviewed excerpts linked to main themes and sub-themes, after reading full transcripts to contextualize those excerpts within the complete narratives. Although the sample size was determined in advance, the three researchers involved in the data analysis agreed that the generated data was adequate, as the replication of themes and comments by participants revealed a high degree of coherence. The results below show all the themes and sub-themes that emerged from interviews with both patients and caregivers.

## Results

### Sample characteristics

Eight patients with chronic DFUs, and six of their caregivers, were included in the study. Participants’ clinical and sociodemographic characteristics are summarized in Table 1. Patients were mainly middle-aged men (the sample included only one woman), while caregivers were younger women.

**Table 1:** Demographic and clinical characteristics of patients with chronic DFUs (*N* = 8) and their caregivers (*N* = 6)

	Patients		Caregivers
	*n* (%) / *M* ± *SD*	Min–Max	*n* (%) / *M* ± *SD*
Gender			
Women	1 (12.5)		6 (100.0)
Men	7 (87.5)		0 (0.0)
Age	56.63 ± 12.01		48.83 ± 8.52
Residence			
Rural	6 (75.0)		5 (83.3)
Urban	2 (25.0)		1 (16.7)
Marital status			
Single	0 (0.0)		1 (16.7)
Married/ non married partnership	5 (62.5)		5 (83.3)
Divorced/ separate	3 (37.5)		0 (0.0)
Education (years)	6.13 ± 2.17		
≤ Primary studies	3 (37.5)		1 (16.7)
≤ Secondary studies	5 (62.5)		5 (83.3)
Professional situation			
Employed	2 (25.0)		1 (16.7)
Unemployed	3 (37.5)		5 (83.3)
Disability pension	3 (37.5)		0 (0.0)
Monthly income			
< 600 €	5 (62.5)		
600 € to 1200 €	2 (25.0)		
> 1200 €	1 (12.5)		
Adequate health literacy			
Yes	3 (37.5)		
No	5 (52.5)		
DM 2 duration (years)	18.63 ± 11.05	5.0–38.0	
HbA1c (%) at the first consultation	9.80 + 2.28	6.7–14.0	
First DFU	4 (50.0)		
DFU duration (weeks)	8.50 + 2.56	6.0–13.0	
Diabetic foot type			
Neuropathic	5 (62.5)		
Neuroischemic	3 (37.5)		
Number of comorbidities^a^			
4	4 (50.0)		
6	4 (50.0)		
Healed DFU after completing the intervention^b^	3 (37.5)		
Although not healed, the DFU improved after completing the intervention^c^	3 (37.5)		
Relationship with the patient			
Wife/ partner			4 (66.7)
Daughter			2 (33.3)
Caregiving duration (years)			10.58 ± 14.78

^a^Comorbidities included diseases such as high blood pressure, dyslipidemia, obesity, diabetic retinopathy, sensorimotor neuropathy, nephropathy, ischaemic heart disease, peripheral arterial disease, among others

^b^healing was defined as the complete epithelization of the wound, assessed through the RESVECH 2.0-PT

^c^DFU improvement was considered when there was a reduction of the wound area

### Patients’ themes and sub-themes

Interviews with all patients yielded four key themes: 1) perspectives regarding the intervention; 2) intervention effectiveness; 3) perceived importance of psychology in the DFU treatment; and 4) emotions and consequences associated with the DFU. Although themes were common to both treatment groups, the last two themes included different sub-themes. Table 2 presents themes, sub-themes, and supporting quotes from patients’ interviews.

**Table 2:** Patient’s themes, sub-themes, and extracts of patients 'sayings

Themes	Sub-themes	Patients’ quotes
Perspectives regarding the intervention	Knowledge about the intervention	TG1: *I have never heard about it* (Male, aged 49)
		TG2: *I did not know about these sessions* (Male, aged 43)
	Usefulness	TG1: *I think it is helpful because sometimes the disease is in the mind* (Male, aged 62)
		TG2: *Patients get other psychological disposition to face the disease* (Male, aged 66)
	Interest in further sessions	TG1: *If it was possible to receive more (sessions), I would attend more* (Male, aged 49)
		TG2: *At the time, I said that I would like to have more because, at that time, it was only four (sessions)* (Female, aged 55)
	Home practice	TG1: *I started trying to do at home what I was doing here* (Male, aged 49)
	Improvement suggestions	TG1: *If it (the intervention) was included in the consultation, it would help to reduce stress* (Male, aged 49)
		TG2: *Sessions should be once a week so that relaxation could last longer* (Female, aged 55)
Intervention effectiveness	Physical changes	TG1: *Because I think it started to heal a little bit more with the relaxation* (Male, aged 49)
		TG2: *I will be honest, while I had the four sessions, it (the wound) improved a lot, a lot* (Male, aged 47)
	Behavioural changes	TG1: *I had the wound, came here for consultations, and since then I stopped drinking* (Male, aged 62)
		TG2: *For example, in the afternoon I was sitting and, when she (her daughter) got home, she would do all the household chores* (Female, aged 55)
	Psychological changes	TG1: *Psychologically, I am better and I believe the wound is going to heal* (Male, aged 80)
		TG2: *After sessions, you feel more peaceful and more confident* (Male, aged 47)
	Interpersonal changes	TlG1: *I was not so aggressive in my daily life. I should say less demanding, and more benevolent at home* (Male, aged 80)
		TG2: *For example, I was not so nervous with the kids. I think I was more patient with the kids* (Female, aged 55)
	Duration of perceived effects	TG1: *For example, when I left sessions, I was calmer for two or three days* (Male, aged 49)
		TG2: *Over two or three weeks, because over two or three weeks period I thought a lot about what was said during sessions* (Male, aged 47)
Perceived importance of psychology in the DFU treatment	Importance of psychology	TG1: *As in all things, the psychological dimension is very important because, if we crash and lose heart, things get worse* (Male, aged 80)
		TG2: *The psychological dimension is very important for things to evolve* (Male, aged 66)
	Psychology related bias	TG1: *In my youth, there was this idea that “I do not need a psychologist, I am not crazy”* (Male, aged 80)
Emotions and consequences associated with DFUs	Fear	TG1: *I was really scared. I never thought this would heal. I* was really afraid (Male, aged 62)
		TG2: *I did not know if it was going to get better or worse, if they had to cut my foot. Today, I am still afraid of that because here they do not inform us of anything* (Male, aged 43)
	Sadness	TG1: *Because, when I dwelt on that I was bad, I got worse. Really worse. I could not go shopping, I really could not do anything* (Male, aged 62)
	Revulsion	TG1: *I was disgusted, anguished… I already am an outraged person* (Male, aged 49)
	Impossibility to work	TG1: *And I worked, I never stopped working* (Male, aged 62)
		TG2: *I felt good for a long time after sessions. Yet, I did a lot of work considering I was a woman with a wounded foot* (Female, aged 55)

*TG1* Treatment group 1 (progressive muscle relaxation with guided imagery), *TG2* Treatment group 2 (hypnosis with guided imagery)

### Perspectives regarding the intervention

Patients had no previous contact with PMR + GI and H + GI sessions. Although one patient from TG1 knew generally what muscle relaxation sessions consisted of, none of TG2 patients had previous knowledge regarding these sessions. All patients from both groups reported sessions were satisfying, beneficial, or important for patients with DFUs. In TG1, patients stressed the importance of sessions for patients’ wellbeing, and as a complement to the medical treatment, while TG2 patients emphasized the sessions capacity to “calm the mind”, change the way of thinking, and help to accept the complexity of the DFU healing.

Two patients from TG1 and three from TG2 expressed their interest in receiving more sessions. In TG1, patients also reported practicing PMR + GI exercises at home, on their own initiative. Most patients suggested that both interventions should include more sessions, and that the number of sessions should be defined according to an initial personalized evaluation. Patients from TG2 also commented that the sessions should have been implemented in a more private room, with no interruptions, and that H + GI intervention should be included in the hospital standard DFU treatment.

### Intervention effectiveness

Patients from both treatment groups reported physical, psychological, behavioural, and interpersonal changes as a result of stress reduction interventions. All participants, except one from TG1, reported perceived improvements in the DFU evolution. Other physical improvements were noticed by patients from TG1, such as better blood circulation, ability to walk, and body balance. Patients from TG2  reported better glycemic control, less pain, and more breathing control.

Psychological changes perceived by patients included feeling calm, weightless or relaxed, and more positive thinking, as reported by three patients from TG1 and the totality from TG2. In both groups, patients also mentioned improvements regarding disease adaptation and sleep quality.

Participants from both groups noticed improvements related tbehavioural o interventions, specifically regarding adherence to self-care. One patient from TG1 also reported a decrease in his alcohol addiction problem, which he considered to be an indirect outcome of the intervention. All patients from TG2, and three patients from TG1, reported changes in their interpersonal relationships as they felt they were more patient and less offensive at home and with other close persons.

According to one patient from TG1, sessions had an impact that lasted about two or three days, and later started to progressively decrease. Other patient from TG2 also expressed that the effect only lasted during the intervention sessions. Two patients, one from each group, referred that sessions had a prolonged effect after the complete treatment. Finally, two other participants from the TG2 reported that the effect lasted from one session to another (three weeks) and that, even today, they still feel the impact.

### Perceived importance of psychology in the DFU treatment

Three patients from TG1, and two from TG2, stressed the importance of psychological interventions for the DFU treatment, and mentioned the patient psychological state as essential for the DFU healing process. Three patients from TG1 reported that many patients may decline psychological interventions due to prejudice related with psychological support.

### Emotional consequences associated with DFUs

Patients expressed some negative feelings associated with DFUs. In TG1, feelings such as sadness, revulsion, and fear of amputation were identified, while, in TG2, fear of amputation, distress, or trauma was mentioned. In both groups, most patients described their daily  life when they were actively working and socially productive, emphasizing how important the professional dimension was to them, and how that changed after the emergence of a DFU.

### Caregivers’ themes and sub-themes

Interviews with family caregivers resulted in four main themes shared by both groups, although with some differences in the fourth theme. Caregivers’ four themes were similar to the ones found with patients, described above. Themes, sub-themes, and extracts of caregiver's sayings are presented in Table 3.

**Table 3:** Caregiver’s themes, sub-themes, and extracts of carevivers' sayings

Themes	Sub-themes	Caregiver’s quotes
**Perspectives regarding the intervention**	Knowledge about the intervention	TG1: *In the context of the diabetic foot, I was told by my father about these sessions* (Caregiver 2, aged 43)
		TG2: *I know very little* (Caregiver 1, aged 36)
	Usefulness	TG1: *These sessions made him good. He got better, although I did not noticed a big difference* (Caregiver 1, aged 49)
		TG2: *I think that it is very helpful because he is a difficult patient and he improved* (Caregiver 2, aged 50)
	Need for more sessions	TG1: *I thought that sessions made him feel good and that he needed more* (Caregiver 3, aged 55)
		TG2: *If he continued (the sessions), I think he would have improved even more his health because he has a lot of pain* (Caregiver 2, aged 50)
	Improvement suggestions	TG1: *It was four sessions, they were not many* (Caregiver 1, aged 48)
		TG2: *But I think that these sessions should be offered to him and other patients at the beginning (of treatment)* (Caregiver 2, aged 50)
**Intervention effectiveness**	Physical changes	TG1: *He told me that he noticed his blood pressure was lower* (Caregiver 2, aged 43)
		TG2: *Now, the wound is healing a little bit, but it has been really worse* (Caregiver 1, aged 36)
	Behavioural changes	TG1: *For example, he used to smoke and eat everything and now he does not. He used to eat pastries and drink coffees* (Caregiver 4, aged 60)
		TG2: *Now, she does not do many of the things she did before. For example, we have a field and since this heel wound appeared she did not work there anymore* (Caregiver 1, aged 36)
	Psychological changes	TG1: *I felt he was calmer, patient, more receptive* (Caregiver 1, aged 49)
		TG2: *He is calmer! He has more patience. When he leaves this place, he goes more relaxed, and he is not always muttering* (Caregiver 2, aged 50)
	Interpersonal changes	TG1: *He is much better. Even with the children. My kids tell me “He has changed so much!”* (Caregiver 4, aged 60)
		TG2: *In the ambulance, he does not complaint anymore with the firemen* (Caregiver 2, aged 50)
	Duration of perceived effects	TG1: *Since sessions ended I think his mood got worse* (Caregiver 3, aged 55)
		TG2: *In the next days he was well. Even today, I notice some changes* (Caregiver 2, aged 50)
**Perceived importance of psychology in the DFU treatment**		TG1: *I believe that people’s psychological state helps in all aspects for their recovery* (Caregiver 2, aged 43)
		TG2: *Because if I cut one finger, even if my family tries to support me, it is not the same thing as having a psychologist* (Caregiver 2, aged 50)
**Emotions and consequences associated with DFUs**	Fear and suffering	TG1: *He is afraid of having to amputate his foot. I told him they will amputate only as a last resort* (Caregiver 3, aged 55)
		TG2: *At times, she went to the consultation and she was told that things were not going well. They scared her* (Caregiver 1, aged 36)
	Patient’s routine	TG1: *He is all day watching TV or in Facebook* (Caregiver 4, aged 60)
	Patient’s unemployment	TG1: *He is off work due to sick leave and he was used to work every day, even on Saturdays* (Caregiver 3, aged 55)
		TG2: *Because a lot of patients have a job and have to support their families, and they start to think how will they support their families, right?* (Caregiver 2, aged 50)
	Caregiver’s social activities	TG1: *I have to stay at home all day. On weekends, who does not want to take a walk?* (Caregiver 4, aged 60)
		TG2: *It is just me and him, and we cannot go out, right? (…) We do not have much interaction with others* (Caregiver 2, aged 50)

*TG1* Treatment group 1 (progressive muscle relaxation with guided imagery), *TG2* Treatment group 2 (hypnosis with guided imagery)

### Perspectives regarding the intervention

One caregiver from TG1 said she knew muscle relaxation sessions, but none of the remaining caregivers had previous knowledge about the interventions. All caregivers considered that sessions were very useful and an important complement to the standard medical treatment for DFU, especially because they notice some differences in their family member.

Two caregivers from TG1 and one caregiver from TG2 reported that the patient needed more sessions. Three caregivers from TG1 commented that if sessions were implemented in a non-hospital setting, and the protocol included more sessions, the intervention would be more effective. According to these caregivers, the hospital environment may not be favorable to relaxation interventions, and a non-hospital setting may, therefore, be more appropriate. Caregivers also considered that more than four sessions could optimize the positive effect on patients’ outcomes. One caregiver from TG2 suggested that sessions should be offered before the patient’ first amputation in order to be more useful.

### Intervention effectiveness

Caregivers also described physical, psychological, behavioural, and interpersonal changes as a result of stress reduction interventions. All caregivers, except one from TG2, reported improvements in the DFU healing. One caregiver from TG1 also referred improvements in the blood pressure, while other caregiver from TG2 said she noticed improvements in the patient’s foot pain.

Three caregivers of patients who received the relaxation intervention reported that their family member was calmer, relaxed, and pacified. One of the caregivers of patients who received the hypnosis intervention also noticed that her partner was calmer, more patient and happier, being less demanding or grumpy. Regarding behavioural changes, one caregiver from TG1 reported that her husband had stopped drinking, smoked less, and improved his diet while receiving sessions. Similarly, another caregiver said that, after completing the hypnosis sessions, her mother adopted more self-care behaviors such as resting her feet, and avoiding the agricultural work. Three caregivers from TG1 reported family members becoming more tolerant and considerate with them, and one caregiver from TG2 stated her partner was much calmer when he had to wait for the diabetic consultations, the ambulance, or even in the supermarket line.

Regarding the duration effects, two accounts from TG1 indicated that the effect stayed over time, however, after the end of the intervention, the impact started to disappear. Nevertheless, one caregiver from TG2 reported that the intervention effect was still visible, at present, two weeks after completing the intervention.

### Perceived importance of psychology in the DFU treatment

Caregivers from both groups considered that the patients’ psychological state was determinant to the treatment success. In TG1, caregivers stressed determination and positive thinking, while one caregiver from TG2 highlighted the importance of psychology to help patients accept and adapt to the disease.

### Emotions and consequences associated with DFUs

In both groups, caregivers mentioned the fear of amputation patients felt every time they went to a consultation, in part due to a lack of information shared by the medical team. According to caregivers from TG1, the DFU had a significant impact on patients’ routine, resulting in patients’ inactivity, isolation, and depression. In both groups, caregivers reported that patients’ unemployment and lack of social activities emerged as a consequence of having a DFU.

## Discussion

Thematic analysis of participants’ experiences revealed that all patients and caregivers who accepted to participate in this study were satisfied, and shared positive opinions about the effectiveness of the two stress reduction interventions on the patient’s psychological wellbeing and DFU healing progression. Patients were mainly men, on average with 57 years old, and professionally inactive, while their caregivers were younger women, mostly their spouses, unemployed, caring for their relative, in average, for 11 years. This demographic characterization was expected since it is in accordance with previous descriptions of patients with DFUs and their caregivers described in national and international studies [36].

Interviews with patients yielded four key themes (common to caregivers): (1) perspectives on the intervention, (2) intervention effectiveness, (3) perceived importance of psychology in the DFU treatment, and (4) emotions and consequences associated with DFUs. Regarding their perspectives on interventions, patients had never experienced relaxation or hypnosis. They reported that interventions’ sessions were satisfactory, beneficial, and important because it improved their feeling of wellbeing as an adjuvant to medical treatment, and helped them to accept and adapt to living with a DFU. Interventions were well received by patients and perceived as effective by caregivers, indicating a high level of acceptability, which might support the adherence to this type of interventions if available in hospital settings/contexts.

Patients and caregivers made some suggestions to improve the implementation of this type of interventions as an adjuvant to medical treatment. All patients suggested that more sessions should be offered, proposing that the number of sessions should be defined according to the patient’s needs, based on an initial assessment. Patients also suggested that sessions should be implemented in a more private space. Similarly, caregivers recommended interventions to include more sessions, suggesting that they should be implemented in a non-hospital setting. Caregivers also emphasized the importance of the interventions to be offered in an initial phase of medical treatment, and before the patient’s amputation. These suggestions are useful since they reflect the perspectives and needs of patients with DFUs and their family caregivers. In fact, patient and public involvement in healthcare provision, specifically in the design, conduct and dissemination of healthcare innovation services (such as the inclusion of stress reduction interventions in the DFUs treatment) is becoming more common in advanced health care systems [37].

Half of patients who received PMR + GI reported practicing the intervention exercises at home, on their own initiative, thus presenting greater adherence in between sessions. One of those patients perceived longer lasting effects of relaxation, compared to a patient that reported having difficulties with the breathing exercises. Patients who received H + GI claimed that those sessions should be included in standard treatment for DFU, which is supported by previous research validating the beneficial clinical impact of hypnosis [38]. A substantial body of research has revealed the efficacy of hypnosis as part of the integrative treatment of many conditions that traditional medicine has found difficult to treat [39]. In fact, hypnosis has shown not only to reduce anxiety in medical conditions but also to change physiological parameters [40], being effective in the management of diabetes, including the regulation of blood sugar [23]. Although, so far, no studies have shown the effectiveness of hypnosis in accelerating DFU healing, in this study, participants’ perceptions suggest that sessions have been helpful to patients with DFUs. Nevertheless, perceived improvements by participants are subjective and were not objectively assessed by researchers.

Patients and caregivers from both groups perceived physical, behavioural, psychological, and interpersonal changes, associated with interventions, highlighting the benefits of stress reduction sessions in patients with DFUs [22, 24, 25, 41]. Considering that studies have shown that stress management improves diabetes [42], participants’ perceptions of physical improvements, such as less pain and better glycemic control, make intuitive sense.

Patients reported several emotional changes/ improvements (e.g., feeling calm, positive thinking, acceptance of the disease) that were also noticed by their caregivers. In fact, psychological interventions don’t only have positive effects in reducing negative emotions, but also may promote the development of a cognitive and emotional process of diabetes acceptance as a chronic disease, thus helping patients to cope with it.

Behavioural changes perceived by participants as an effect of interventions were associated with adherence to self-care behaviors. One patient reported a decrease in alcohol and tobacco consumption, as well as the adoption of a healthier diet, which he and his caregiver associated with the relaxation sessions. Patients also reported being more patient and less offensive, which was also corroborated by caregivers. Foot ulcers in people with diabetes are associated with high levels of morbidity, with symptoms of anxiety and depression being the most prevalent [8, 9, 43, 44]. Therefore, it makes sense that psychological interventions may have a positive effect and an indirect impact on medication adherence, empowering patients to engage in self-care behaviors, and boosting overall mood [43], as suggested by this study’s participants.

Regarding the duration of the changes resulting from interventions, participants’ opinions differed in both groups. They ranged from effects that only lasted during the session to longer-term effects, after the end of the interventions. TG2 patients and caregivers reported longer effects, as most of them expressed that the effect remained over time, and were still visible two weeks after completing the intervention. In fact, the use of hypnosis has been found to promote positive changes, with longer lasting effects. For example, previous studies have shown hypnosis as a promising therapeutic complementary intervention to reduce impulsive behaviors, over time, in obese patients [45]. Wood and colleagues [46] also showed that the hypnotic intervention altered T-cell activity what may explain the longer effects hypnosis may have on healing. Nevertheless, in this study, the duration of perceived effects were limited in time, especially in TG1, as most participants reported that effect started to vanish after the sessions.

In this study, most patients referred the importance of psychological interventions for the DFU treatment - reflecting a belief in the mind-body connection – although some patients may feel reluctant to participate in psychological interventions due to prejudice or shame, or even because they feel emotionally overwhelmed by the consequences of the disease. Therefore, psychological interventions should be available in an early period of the DFU diagnosis [43]. Caregivers also stressed the role of psychological status for successful treatments, determination, positive thinking, and acceptance of the disease, highlighting caregivers’ awareness of the importance of psychological intervention to help the patient accept the disease [47].

Patients, especially those from TG1, reported that DFUs were a source of negative emotional consequences, such as sadness, anger, revulsion, and anguish, living with the fear of amputation and trauma [8, 9, 44], and dealing with the impossibility to work. Caregivers from both intervention groups stressed the fear of amputation felt by patients. As previously suggested in the literature [48], some individuals with diabetes fear amputation worse than death. Given these negative emotions, the role of psychological interventions may be helpful to improve patient’s general wellbeing, reduce symptoms of anxiety and depression, and stimulate emotional regulation [43], particularly when patients are unemployed, inactive, and with their QoL compromised due to DFUs [44].

Regarding consequences associated with DFUs, caregivers highlighted the impact on the patient’s daily life, resulting in inactivity, isolation, depression, unemployment, and lack of social activities for the caregiver, as shown in previous studies [14, 15]. The scenario of burden appears to be exacerbated following amputation [49]. In this sense, one of the caregivers believed that stress interventions might be more beneficial before amputation surgery.

Overall, the aim of this study was to understand whether the two stress reduction interventions (PMR + GI and H + GI) had an impact on psychological factors that have been reported to have a negative effect on wound healing [50, 51]. According to participants’ perceptions, psychological interventions had a positive effect on patients’ behavioural, emotional, and interpersonal dimensions, being also associated with perceived DFU’s improvements, and reduced symptoms of psychological morbidity. These results suggest the potential positive effects of both interventions on patients’ emotional state, ulcer healing, and general wellbeing, as perceived by patients and family caregivers.

### Limitations

Despite the promising findings, this study has some limitations that deserve attention. This study was based on the subjective perceptions of a reduced number of participants and, as such, they need to be interpreted cautiously. Although the thematic analysis of the interviews indicated a level of coherence regarding the emerging themes, the inclusion of more participants would benefit future studies. The purposeful sampling has the potential for bias in recruitment creating a possible influence of confounders that were not controlled. All psychologists who performed the stress reduction techniques were highly trained, but there might have been bias regarding the person of the therapist and the therapist ‘s gender that was not controlled for. The non-inclusion of a group of patients with no intervention, and their respective caregivers, does not allow to determine a relationship between the intervention and the outcomes, i.e. if the perceived improvements reported by participants would also been identified by participants that did not receive any stress reduction intervention. Finally, only patients from two hospitals in the north of Portugal were involved. Therefore, future research is needed to better understand the impact of stress reduction techniques on DFU healing and psychological wellbeing.

### Implications for clinical practice

This study provides promising data supporting the benefits of stress reduction interventions, relaxation and hypnosis, for clinically distressed patients with DFUs. If further research confirms this study’s findings, both interventions should be included as standard treatments for patients with DFUs in addition to clinical/medical treatment. Muscle relaxation interventions may be conducted by trained clinical and health psychologists that already work in the hospital, not requiring additional financial efforts. Hypnosis sessions are conducted by trained professionals in hypnotherapy, which may require some initial financial investment. However, both intervention techniques may be easily taught to patients so that they can practice self-relaxation and self-hypnosis exercises at home. To promote home practice on a daily basis, sessions may be recorded and made available to patients who should be coached in self-relaxation and self-hypnosis, using a taped script or a smartphone application. According to participants’ suggestions, psychological interventions should be available early on, when the patient begins treatment, i.e. in the first diabetic foot consultation. Thus, a stress reduction protocol that would include a careful psychological evaluation, which is common practice in other chronic diseases/ conditions, would allow clinically distressed patients’ referral to an individual/ group stress reduction intervention.

Considering patients and caregivers’ perceptions regarding DFUs healing and psychological wellbeing, during and after intervention, it would be interesting to further evaluate the benefits of implementing a psychological support/consulting service for patients with DFUs in multidisciplinary diabetic foot clinics. Distressed caregivers may also be offered a support group to help reduce overload, especially among caregivers who care for patients who suffered an amputation, have a chronic illness, report physical symptoms, and have been caregivers for several years [49].

Future studies should address the patient-caregiver dyad, over time, and better understand how relaxation and hypnosis promote QoL, adherence to medical treatment, and self-care behaviors. This would allow the creation of a psychological intervention protocolto answer patients’ needs, as well the needs of informal caregivers, and the health professional team caring for patients with DFUs.

## Conclusion

The goal of the stress reduction interventions was to increase psychological wellbeing and, consequently, promote the conditions that facilitate DFU healing. Overall, patients and caregivers were satisfied with both types of psychological interventions, and perceived several improvements as a result of sessions. This study provides promising indications regarding the benefits of both interventions for clinically distressed patients with DFUs, however, further research on the effectiveness of interventions is required to strengthen the findings of the present study. The deeper understanding of patients and caregiver’s perspective on stress reduction interventions as adjuvant to standard medical treatment may also shed light on the mechanisms that are involved in the relationship between psychological stress, physiological stress, and DFU healing.

## Data Availability

Requests for further details on the datasets can be submitted to the corresponding author (gracep@psi.uminho.pt).

## Abbreviations

DFU: Diabetic foot ulcer
DM: Diabetes mellitus
H+GI: Hypnosis with guided imagery intervention
HADS: Hospital Anxiety and Depression Scale
PMR+GI: Progressive muscle relaxation with guided imagery intervention
PSS: Perceived Stress Scale
QoL: Quality of life
RCT: Randomized control trial
TG1: Treatment group 1
TG2: Treatment group 2

## References

[1] International Diabetes Federation (2021). IDF Diabetes Atlas.

[2] Shin L, Armstrong D, Sanders L (2020). Foot Ulcers. Johns Hopkins Diabetes Guide.

[3] Armstrong DG, Boulton AJM, Bus SA (2017). Diabetic foot ulcers and their recurrence. N Engl J Med.

[4] Lipsky BA, Senneville E, Abbas ZG, Aragón-Sánchez J, Diggle M, Embil JM, Kono S, Lavery LA, Malone M, van Asten SA, Urbančič-Rovan V, Peters E, International Working Group on the Diabetic Foot (IWGDF) (2020). Guidelines on the diagnosis and treatment of foot infection in persons with diabetes (IWGDF 2019 update). Diabetes Metab Res Rev.

[5] Musa HG, Ahmed ME (2012). Associated risk factors and management of chronic diabetic foot ulcers exceeding 6 months’ duration. Diabet Foot Ankle.

[6] Armstrong DG, Swerdlow MA, Armstrong AA, Conte MS, Padula WV, Bus SA (2020). Five year mortality and direct costs of care for people with diabetic foot complications are comparable to cancer. J Foot Ankle.

[7] Edmonds M, Manu C, Vas P (2021). The current burden of diabetic foot disease. J Clin Orthop Trauma.

[8] Khunkaew S, Fernandez R, Sim J (2019). Health-related quality of life among adults living with diabetic foot ulcers: a meta-analysis. Qual Life Res.

[9] Polikandrioti M, Vasilopoulos G, Koutelekos I, Panoutsopoulos G, Gerogianni G, Babatsikou F, Zartaloudi A, Toulia G (2020). Quality of life in diabetic foot ulcer: associated factors and the impact of anxiety/depression and adherence to self-care. Int J Low Extrem Wounds.

[10] Roy T, Lloyd CE (2012). Epidemiology of depression and diabetes: a systematic review. J Affect Disord.

[11] Jiang FH, Liu XM, Yu HR, Qian Y, Chen HL (2022). The incidence of Depression in Patients with Diabetic Foot Ulcers: a systematic review and Meta-analysis. Int J Low Extrem Wounds.

[12] Ahmad A, Abujbara M, Jaddou H, Younes NA, Ajlouni K (2018). Anxiety and depression among adult patients with Diabetic Foot: Prevalence and Associated factors. J Clin Med Res.

[13] Messenger G, Taha N, Sabau S, AlHubail A, Aldibbiat AM (2019). Is there a role for informal caregivers in the management of diabetic foot ulcers? A narrative review. Diabetes Ther.

[14] Hançerlioğlu S, Toygar İ, Ayhan A, Yilmaz İ, Orhan Y, Özdemir GS, Şimşir IY, Çetinkalp Ş (2021). Burden of Diabetic Foot Patients’ caregivers and affecting factors: a cross-sectional study. Int J Low Extrem Wounds.

[15] Nabuurs-Franssen MH, Huijberts MSP, Nieuwenhuijzen Kruseman AC, Willems J, Schaper NC (2005). Health-related quality of life of diabetic foot ulcer patients and their caregivers. Diabetologia.

[16] Costa MS, Machado JC, Pereira MG (2020). Longitudinal changes on the quality of life in caregivers of type 2 diabetes amputee patients. Scand J Caring Sci.

[17] Suglo JN, Winkley K, Sturt J (2022). Prevention and Management of Diabetes-Related Foot Ulcers through Informal Caregiver involvement: a systematic review. J Diabetes Res.

[18] Gottrup F, Apelqvist J (2012). Present and new techniques and devices in the treatment of DFU: a critical review of evidence. Diabetes Metab Res Rev.

[19] Rigor J, Martins-Mendes D, Monteiro-Soares M. Risk factors for mortality in patients with a diabetic foot ulcer: a cohort study. Eur J Intern Med. 2020. 10.1016/j.ejim.2019.11.011. 10.1016/j.ejim.2019.11.011 31735544

[20] Iversen MM, Tell GS, Espehaug B, Midthjell K, Graue M, Rokne B, Berge LI, Østbye T (2015). Is depression a risk factor for diabetic foot ulcers? 11-years follow-up of the Nord-Trøndelag Health Study (HUNT). J Diabetes Complications.

[21] Pearson S, Nash T, Ireland V (2014). Depression symptoms in people with diabetes attending outpatient podiatry clinics for the treatment of foot ulcers. J Foot Ankle Res.

[22] Deswita D, Sahar J, Mulyono S (2019). Impact of coaching and self-hypnosis intervention on blood glucose levels of older adults in Indonesia. Enferm Clin.

[23] Rodrigues F, Oliveira C, Silva CF, D’Almeida A (2017). Psychotherapy with hypnosis in glycemia in patients with type 1 diabetes Mellitus. EpSBS.

[24] Xu Y, Cardeña E (2007). Hypnosis as an Adjunct Therapy in the management of diabetes. Int J Clin Exp Hypn.

[25] Rice BI, Kalker A, Schindler J, Dixon RM (2001). Effect of biofeedback-assisted relaxation training on foot ulcer healing. J Am Podiatr Med Assoc.

[26] Pereira MG, Vilaça M, Carvalho E (2022). Effectiveness of two stress reduction interventions in patients with Chronic Diabetic Foot Ulcers (PSY-DFU): protocol for a longitudinal RCT with a nested qualitative study involving Family Caregivers. Int J Environ Health Res.

[27] Guetterman TC, Fetters MD, Creswell JW (2015). Integrating quantitative and qualitative results in health science mixed methods research through joint displays. Ann Fam Med.

[28] Kuhnke JL, Bailey PH, Woodbury MG, Burrows M (2014). The role of qualitative research in understanding Diabetic Foot Ulcers and Amputation. Adv Skin Wound Care.

[29] Zigmond AS, Snaith RP (1983). The hospital anxiety and depression scale. Acta Psychiatr Scand.

[30] Pais-Ribeiro J, Silva I, Ferreira T, Martins A, Meneses R, Baltar M (2007). Validation study of a portuguese version of the hospital anxiety and Depression Scale. Psychol Health Med.

[31] Cohen S, Williamson G, Spacapan S, Oskamp S (1998). Perceived stress in a probability sample of the United States. The social psychology of health: claremont symposium on applied social psychology.

[32] Trigo M, Canudo N, Branco F, Silva D (2010). Estudo das propriedades psicométricas da perceived stress scale (PSS) na população Portuguese [Psychometric proprieties of the perceived stress scale (PSS) in portuguese population]. Psychologica.

[33] Van Rijnsoever FJ. (I Can’t get no) saturation: a simulation and guidelines for sample sizes in qualitative research. PLoS One. 2017. 10.1371/journal.pone.0181689. 10.1371/journal.pone.0181689 PMC5528901 28746358

[34] Kiger ME, Varpio L. Thematic analysis of qualitative data: AMEE Guide No. 131. Med Teach. 2020. 10.1080/0142159X.2020.1755030. 10.1080/0142159X.2020.1755030 32356468

[35] Miles MB, Huberman AM (1994). Qualitative data analysis.

[36] Pedras S, Carvalho R, Pereira MG (2014). Sociodemographic and clinical characteristics of patients with diabetic foot ulcer. Rev Assoc Med Bras.

[37] Cluley V, Ziemann A, Feeley C, Olander EK, Shamah S, Stavropoulou C (2022). Mapping the role of patient and public involvement during the different stages of healthcare innovation: a scoping review. Health Expect.

[38] Yeh VM, Schnur JB, Montgomery GH (2014). Disseminating hypnosis to health care settings: applying the RE-AIM framework. Psychol. Conscious.

[39] Elkins G, Jensen MP, Patterson DR (2007). Hypnotherapy for the management of chronic pain. Int J Clin Exp Hypn.

[40] Weisberg MB (2008). 50 years of hypnosis in medicine and clinical health psychology: a synthesis of cultural crosscurrents. Am J Clin Hypn.

[41] Pereira MG (2017). Changing the mind: hypnosis and diabetes. Rev Lat Am Enfermagem.

[42] Surwit RS, Van Tilburg MA, Zucker N, McCaskill CC, Parekh P, Feinglos MN, Edwards CL, Williams P, Lane JD (2002). Stress management improves long-term glycemic control in type 2 diabetes. Diabetes Care.

[43] Akyirem S, Forbes A, Ward JL, Due-Christensen M (2022). Psychosocial interventions for adults with newly diagnosed chronic disease: a systematic review. J Health Psychol.

[44] Pedras S, Carvalho R, Pereira MG (2018). Predictors of quality of life in patients with diabetic foot ulcer: the role of anxiety, depression, and functionality. J Health Psychol.

[45] Roslim NA, Ahmad A, Mansor M, Aung MMT, Hamzah F, Hassan H, Lua PL (2021). Hypnotherapy for overweight and obese patients: a narrative review. J Integr Med.

[46] Wood GJ, Bughi S, Morison J, Tanavoli S, Tanavoli S, Zadeh H (2003). Hypnosis, differential expression of cytokines by T-cell subsets, and the hypothalamo-pituitary adrenal axis. Am J Clin Hypn.

[47] Deter HC (2012). Psychosocial interventions for patients with chronic disease. Biopsyhcosoc Med.

[48] Wukich DK, Raspovic KM, Suder NC (2018). Patients with diabetic foot disease, fear major lower-extremity amputation more than death. Foot Ankle Spec.

[49] Costa S, Machado JC, Pereira MG (2018). Longitudinal burden changes for caregivers of patients with type 2 diabetes: a longitudinal study. J Adv Nurs.

[50] Ebrecht M, Hextall A, Kirtley L, Taylor A, Dyson M, Weinman J (2004). Perceived stress and cortisol levels predict speed of wound healing in healthy male adults. Psychoneuroendocrinology.

[51] Walburn J, Vedhara K, Hankins M, Rixon L, Weinman J. Psychological stress and wound healing in humans: a systematic review and meta-analysis. J Psychosom Res. 2009. 10.1016/j.jpsychores.2009.04.002. 10.1016/j.jpsychores.2009.04.002 19686881

